# Resource Allocation and Trajectory Planning in Integrated Sensing and Communication Enabled UAV-Assisted Vehicular Network

**DOI:** 10.3390/s25237295

**Published:** 2025-11-30

**Authors:** Mingyang Song, Wenyang Zhang, Jingpan Bai

**Affiliations:** 1 School of Computer & Information Engineering, Anyang Normal University, Anyang 455000, China; mingyangsong@whut.edu.cn (M.S.); 13721720629@163.com (W.Z.); 2 School of Computer Science, Yangtze University, Jingzhou 430023, China; 3 Vehicle Measurement, Control and Safety Key Laboratory of Sichuan Province (No. QCCK2025-006), Chengdu 610039, China; 4 Yunnan Key Laboratory of Service Computing, Yunnan University of Finance and Economics, Kunming 650000, China; 5 Hubei Key Laboratory of Oil and Gas Drilling and Production Engineering, Yangtze University, Wuhan 430100, China

**Keywords:** integrated sensing and communication, UAV-assisted vehicle network, resource allocation, trajectory planning, block coordinate descent

## Abstract

**Highlights:**

**What are the main findings?**

- A trajectory design and resource allocation (TDRA) strategy is proposed for ISAC-enabled UAV-assisted vehicular networks, jointly optimizing UAV trajectory, vehicle association, and subchannel allocation.
- The proposed BCD–SCA-based iterative algorithm achieves significant performance gains, with a notable improvement in the average achievable rate compared to benchmarks.

**What is the implication of the main finding?**

- Our method ensures reliable communication and radar sensing services in congestion scenarios by effectively balancing spectrum resources.
- The strategy provides a practical tool for deploying UAV-assisted ISAC vehicular networks, supporting high-efficiency and low-latency driving services.

**Abstract:**

This paper investigates the problem of maximizing the average achievable rate in an unmanned aerial vehicle (UAV)-assisted vehicular network, where UAVs and ground base stations (GBSs) jointly serve vehicular users through integrated sensing and communication (ISAC) technology. To balance communication and sensing performance, we maximize the average achievable rate under radar sensing constraints by jointly optimizing UAV trajectory planning, vehicle association, and subchannel allocation. The resulting problem is a challenging mixed-integer nonlinear program (MINLP) due to the strong coupling among decision variables. To address this, we propose an iterative algorithm based on block coordinate descent (BCD), which decomposes the original problem into three subproblems—vehicle association, UAV trajectory planning, and subchannel allocation—by fixing certain variables. These subproblems are solved alternately using successive convex approximation (SCA) and convex optimization techniques. Simulation results verify the effectiveness of the proposed algorithm, demonstrating superior average achievable rate performance compared with conventional methods under radar sensing constraints.

## 1. Introduction

As Intelligent Transportation Systems (ITSs) advance rapidly, vehicular networks have emerged as a crucial technology for enabling efficient, safe, and sustainable transportation. However, traditional vehicular networks face many challenges in complex urban environments, such as temporary traffic congestion scenarios. Such congestion is typically triggered by sudden events or adverse conditions, including traffic accidents, road construction, bad weather, or peak-hour demand. These factors lead to traffic flow exceeding the road’s capacity, thereby causing congestion. In such scenarios, the rapid growth of vehicles leads to a sharp rise in communication requests within a local area. This heavily burdens the ground network infrastructure and ultimately causes excessive communication load and increased network latency [1].

Recent advances in Unmanned Aerial Vehicle (UAV) technology have introduced novel solutions to address the above problems. UAVs offer high flexibility, rapid deployment, and broad coverage, enabling them to act as mobile aerial nodes that assist vehicular networks and enhance communication efficiency and coverage. By integrating UAVs with a vehicular network, a UAV-assisted Vehicular Network can be formed. Such networks not only alleviate the communication burden on ground infrastructure but also provide vehicles with more reliable communication links and more timely information support. Therefore, UAV-assisted vehicular networks have emerged as a pivotal research focus in the field of vehicular network. Meanwhile, Integrated Sensing and Communication (ISAC) technology has emerged as a pivotal enabler for next-generation wireless systems, attracting widespread attention. ISAC technology enables communication and sensing functions to share the same spectrum resources and hardware modules, which significantly improves spectrum efficiency and reduces resource redundancy [2]. Recent studies further highlight that ISAC can enhance sensing accuracy, provide beyond-line-of-sight perception, and strengthen communication reliability in V2X scenarios [3]. ISAC can expand the sensing range by using communication infrastructures such as Ground Base Stations (GBSs) and UAVs as sensors [4]. For example, in V2X (Vehicle-to-Everything) scenarios, GBSs equipped with integrated ISAC function can be used to expand the sensing range of vehicles. This helps to overcome sensing blind spots and enables vehicles to achieve beyond-line-of-sight perception, thus ensuring driving safety [5].

This paper considers a UAV-assisted vehicular network architecture that integrates ISAC technology. In this architecture, the ISAC is integrated into both UAVs and GBSs. This enables UAVs to cooperate with GBSs to achieve all-round radar sensing of multiple vehicles within the scene. While conducting radar sensing, GBSs and UAVs can simultaneously communicate with surrounding vehicles and share sensing information to enable beyond-line-of-sight perception. However, sharing spectrum resources between communication and radar sensing functions causes mutual influence and trade-offs. If the communication function occupies more spectrum resources, the sensing performance will decrease. Similarly, if the sensing function occupies too many spectrum resources, it will affect the communication performance. Consequently, unreasonable resource allocation may significantly degrade both communication and sensing performance. On the other hand, in temporary traffic congestion scenarios, taller obstructions such as buildings, trees, and overpasses can cause attenuation or blockage of wireless signals, thereby affecting the communication and sensing efficiency between UAVs and vehicles [6,7]. Moreover, the mobility of vehicles can further degrade the communication link quality between UAVs and vehicles, thereby affecting the stability and reliability of communication. Therefore, UAVs that are deployed in fixed positions or have poorly planned flight trajectories cannot effectively provide communication and sensing services to moving vehicles. In summary, jointly optimizing UAV trajectory planning and resource allocation to enhance both communication and sensing performance presents a challenge in the considered architecture. 

To tackle the aforementioned challenges, this paper proposes a Trajectory Design and Resource Allocation (TDRA) strategy. The strategy constructs a resource allocation model for communication and radar sensing. It formulates a joint optimization problem encompassing UAV trajectory planning, vehicle association, and subchannel allocation. This aims to maximize communication rate under the constraints of UAV energy consumption and radar sensing performance. This joint optimization problem is highly attractive in practice. First, reasonable subchannel allocation and function selection enable edge nodes to balance communication and sensing tasks, thereby mitigating the negative effects of spectrum competition between the two functions. Secondly, the optimization of UAV trajectory planning and vehicle association can enhance communication performance, which in turn improves sensing performance. However, the optimization problem mentioned above is intractable. This is because the decision variables of optimization problem are interdependent. This makes the optimization problem complex and difficult to solve analytically. Therefore, a block coordinate descent method-based iterative algorithm is proposed to solve the optimization problem. Specifically, the original optimization problem is divided into following subproblems by fixing some variables: vehicle association, UAV trajectory planning, and subchannel allocation. The original problem’s approximate optimal solution can be obtained through successive alternating optimization of these subproblems. For subproblems, this paper solves them by using the SCA [8] technique and convex optimization methods. The simulation results validate the efficacy of the proposed algorithm. 

The main contributions of this paper can be summarized as follows:

(1) We introduce a UAV-assisted vehicular network architecture that integrates ISAC. In this architecture, UAVs and GBSs equipped with ISAC provide communication services for a group of vehicle users and collaboratively sense targets. Meanwhile, we propose the TDRA strategy, which constructs mathematical models related to communication and radar sensing and establishes an optimization problem. This aims to maximize the communication rate under the constraints of radar sensing performance. 

(2) A sub-optimal method is proposed for solving the optimization problem, which is proven to be a mixed-integer nonlinear optimization problem. Specifically, we partition the optimization problem into three key subproblems: vehicle association, UAV trajectory planning, and subchannel allocation, achieved by fixing a subset of variables. Moreover, we propose a block coordinate descent-based iterative algorithm for alternately solving the subproblems to get the original problem’s approximate optimal solution. For the subproblems in non-convex form, this paper employs the SCA technique to solve them. 

(3) The performance evaluation, conducted via numerical simulations, shows that the proposed algorithm outperforms existing works in terms of average achievable rate. The experimental outcomes confirm that the proposed algorithm is effective. The algorithm converges within a few iterations and achieves near-optimal performance under varying influencing factors, such as the number of subchannels, UAV maximum flight speed, and minimum Mutual Information (MI) requirements. 

The rest of this paper is structured as follows. Section 2 provides an overview of the current state of research both domestically and internationally. In Section 3, the system model is introduced. Section 4 focuses on formulating the optimization problem aimed at maximizing the average achievable rate through the joint optimization of UAV trajectory planning, vehicle association, and subchannel allocation. In Section 5, we propose the communication and sensing resource allocation algorithm and provide the algorithm complexity and convergence analysis. Section 6 presents the simulation results to validate the proposed algorithm. Finally, Section 7 concludes the paper. 

## 2. Related Works

### 2.1. Resource Allocation in ISAC System

Compared with traditional separated radar and communication systems, ISAC achieves higher spectrum efficiency through tightly coupled waveform, hardware, and resource sharing mechanisms [9]. Such spectrum reuse also enables superior sensing–communication tradeoffs in vehicular environments, as reported in recent ISAC–V2X studies [10]. Some researchers have investigated resource allocation strategies in ISAC systems [11,12,13,14,15,16]. These works typically consider ISAC systems that realize communication and sensing functions by sharing spectrum resources. They jointly optimize subchannel allocation and transmit power to maximize communication rate or minimize system power, balancing communication and sensing performance. Zhu et al. [11] proposes a fairness-based communication and sensing resource allocation algorithm. This algorithm assigns subcarriers and power to radar and communication users to facilitate radar sensing and data transmission. Simultaneously, it aims to maximize the rate of the user experiencing the worst channel conditions to ensure fairness among all users. Shi et al. [12] propose a resource allocation strategy that allocates the number of subcarriers for communication and sensing functions and assigns optimal power resources on the corresponding subcarriers to achieve the power consumption minimization in ISAC system. Yang et al. [13] explore the issue of minimizing transmit power in a multi-user ISAC system. The optimization problem considers the joint optimization of subcarrier and power allocation, while also considering network stability and radar detection performance. Wang et al. [14] present an OFDMA-based ISAC system and examine the joint optimization of subchannel and power allocation with the objective of maximizing the communication rate. Zhao et al. [15] proposes an ISAC-based mobile edge computing environment. Meanwhile, a joint device association and subchannel allocation problem is formulated to address the decrease in sensing accuracy and communication capacity caused by resource competition among mobile devices. However, the aforementioned works do not consider the gains that UAVs can bring to communication and sensing performance. Zhang et al. [16] investigates the application of ISAC in multi-UAV collaborative detection scenarios, where UAVs are equipped with ISAC units to perform radar detection and communication functions simultaneously. The paper optimizes UAV transmission power, subchannel allocation, and UAV trajectories to maximize radar detection performance and detection fairness while satisfying the quality demands of communication and sensing. However, this work does not consider the gains that ground edge nodes can bring to communication and sensing performance.

### 2.2. UAV Trajectory Planning

Some works [17,18,19,20,21,22,23,24] have addressed the optimization of latency, energy consumption, and communication rate in UAV-assisted edge computing environments through the joint optimization of UAV trajectory planning, communication, and computation resource management. Zhang et al. [17] formulate a joint optimization problem to maximize UAV service profit in UAV-assisted vehicular edge computing system by considering UAV location, transmit power, computation resources, and vehicle association. Rzig et al. [18] proposes a computation offloading strategy, which maximizes the proportion of successfully offloaded tasks by jointly optimizing the initial flight position, flight direction of UAVs, and vehicle association. Yan et al. [19] investigates offloading strategy in UAV-assisted vehicular network. The strategy jointly optimizes UAV trajectories and task offloading to reduce system latency. Feng et al. [20] designs a communication service provision strategy. This strategy maximizes the total achievable communication rate by jointly optimizing user scheduling and UAV trajectory planning. Nguyen et al. [21] propose a joint optimization problem involving user association, UAV trajectory planning, and subchannel allocation in UAV-assisted wireless networks. Qin et al. [22] investigate the joint optimization problem of UAV trajectory planning, subchannel allocation, and power allocation in a heterogeneous air-ground integrated architecture to maximize system energy efficiency. Singh et al. [23] proposes a UAV-assisted vehicular network architecture, where UAVs act as communication relays to serve vehicles outside the coverage area of GBSs. Wu et al. [24] proposes a UAV-assisted vehicular network architecture for real-time video transmission. The architecture ensures the stability of the communication link between UAVs and vehicles by designing reasonable UAV flight trajectories. However, the aforementioned works do not consider optimizing the allocation of sensing resources, which results in a reduction in the efficiency of providing sensing services. 

Although several prior studies have employed BCD or SCA based iterative methods for UAV-assisted communication or ISAC optimization [11,13,20,21,22], the problem considered in this work is fundamentally different. Existing research does not address cooperative ISAC between UAVs and GBSs, nor the joint coupling among subchannel function selection, MI-based sensing constraints, vehicle–UAV/GBS association, and UAV trajectory planning [17,18,19,20,21]. Furthermore, previous works do not examine how UAV flight trajectories simultaneously influence communication rate and sensing MI in highly dynamic vehicular environments [17,18,19,20,21,22,23,24]. In contrast, the proposed TDRA framework formulates a new optimization problem that integrates dual-function subchannel allocation, mobility-aware access association, and UAV trajectory planning under MI constraints within a unified model. These characteristics create a problem structure that is substantially different from conventional UAV-enabled ISAC formulations and motivate the tailored BCD–SCA algorithm developed in this work.

## 3. System Model

**Figure 1:**
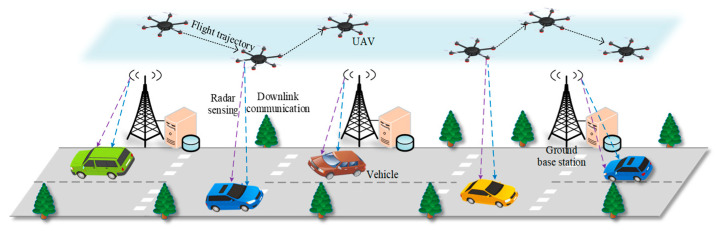
Illustration of a communication and sensing resource allocation scenario.

We consider a communication and sensing resource allocation scenario under a UAV-assisted vehicular network architecture, as shown in Figure 1. The scenario includes *V* vehicles, *R* GBSs, and *U* UAVs. Let VE={1,2,…,V} denote the set of vehicles, RU={1,2,…,R} denote the set of GBSs, and UA={1,2,…,U} denote the set of UAVs. v∈VE, r∈RU, and u∈UA represent any vehicle, GBS, and UAV in their respective sets. Assume that all UAVs and GBSs are equipped with OFDM-based dual-function transmitter [15]. This transmitter can simultaneously achieve millimeter-wave radar sensing and data communication functions by transmitting OFDM signals with different functions, thereby providing radar detection and downlink communication services to the accessing vehicles. That is, the vehicles accessing a UAV or GBS are both the communication targets and radar sensing targets of that UAV or GBS [13]. Assume that the considered network architecture operates in a time-slot manner. Therefore, we divide the time period into *T* equal-length time slots. Let the set of time slots be denoted as $TI=\left\{1,2,\ldots ,T\right\}$, where t∈TI represents any time slot, and the length of each time slot is a fixed value of τ seconds.

Although UAV-assisted vehicular networks may experience Doppler shifts caused by the mobility of both UAVs and vehicles, the Doppler levels in the considered environment fall within the tolerance range of standardized OFDM-based V2X systems, where the Doppler spread remains significantly smaller than the subcarrier spacing adopted in NR-V2X and LTE-V2X [25,26]. Therefore, OFDM does not suffer from severe inter-carrier interference under the operating conditions of this study. While orthogonal time–frequency space (OTFS) modulation offers improved Doppler robustness in rapidly time-varying channels [27], integrating OTFS into UAV-assisted vehicular ISAC is left for future work, as this paper focuses on trajectory–association–resource optimization under the widely deployed OFDM framework.

Integrating ISAC modules into UAV platforms allows UAVs to simultaneously perform radar sensing and downlink data transmission, improving both sensing range and communication coverage, as validated in UAV–ISAC literature [28].

The three-dimensional Cartesian coordinate system is used to denote the positions of vehicles, GBSs, and UAVs. UAVs are deployed in the scene with adjustable horizontal positions and fixed flight altitudes. Let the variable $CU=\left\{CU_{u}^{t}\right\}$ represent the UAV trajectory decision, where $CU_{u}^{t}=\left\{x_{u}^{t},y_{u}^{t}\right\}$ denotes the horizontal position of UAV *u* at time slot *t*, $x_{u}^{t}\in \left[0,x_{\max}\right]$ and $y_{u}^{t}\in \left[0,y_{\max}\right]$ represent the *x* and *y* coordinate values of UAV *u* in the scene, respectively. Since the flight speed of the UAV is limited, the flight speed of UAV *u* at time slot *t* is subject to the following constraint:

(1) $${\left\|CU_{u}^{t}-CU_{u}^{t-1}\right\|}^{2}\leq {\left(md_{u}^{\max}\cdot \tau \right)}^{2},\forall u\in UA,t\in TI$$

where $md_{u}^{\max}$ denotes the maximum flight speed of UAV *u*. 

Assuming that the UAV departs from a starting point, flies for a time period to serve vehicle users, and then needs to reach a preset destination to replenish energy and prepare for the next cycle of service, the UAV’s flight position is constrained by the following conditions:

(2) $$CU_{u}^{1}=POS_{u}^{ini},CU_{u}^{T}=POS_{u}^{fin},\forall u\in UA$$

where $POS_{u}^{ini}=\left(x_{u}^{ini},y_{u}^{ini}\right)$ and $POS_{u}^{fin}=\left(x_{u}^{fin},y_{u}^{fin}\right)$ denote the takeoff and landing positions of UAV *u*’s flight, respectively.

Let $sp_{u}$ denote the energy consumed by UAV *u* per unit distance of flight. Hence, the flight energy consumption of UAV *u* at time slot *t* is given by:

(3) $$FE_{u}^{t}=\left\|CU_{u}^{t}-CU_{u}^{t-1}\right\|\cdot sp_{u}$$

The energy consumed by UAV *u* during the flight over the time period *TC* can be formulated as:

(4) $$EC_{u}^{TC}=\sum_{t=1}^{T}FE_{u}^{t}$$

Considering the limited on-board energy of the UAV, the energy consumption of UAV *u* over the time period *TC* is subject to the following constraint:

(5) $$EC_{u}^{TC}\leq EC_{u}^{\max},\forall u\in UA$$

where $EC_{u}^{\max}$ denotes the on-board energy of UAV *u*.

If the flight distance between UAVs is too close, it may lead to collisions. To ensure flight safety, UAVs need to maintain a certain safe distance:

(6) $${\left\|CU_{u}^{t}-CU_{u^{\prime }}^{t}\right\|}^{2}\geq d_{\min}^{2},\forall u\in UA,u^{\prime }\in UA,u\neq u^{\prime },t\in TI$$

where $d_{\min}$ denotes the minimum safe distance required between UAVs. 

Assuming that vehicles move along fixed routes in the scene, let $CO_{v}^{t}=\left\{x_{v}^{t},y_{v}^{t}\right\}$ denote the horizontal coordinates of vehicle v∈VE at time slot *t*, and $CO_{r}=\left\{x_{r}^{t},y_{r}^{t}\right\}$ represent the fixed position of GBS *r*. Since GBSs and vehicles are located on the ground, this paper assumes that their altitude is 0. Let the distance between UAV *u* and vehicle *v* be denoted as $d_{u,v}^{t}$, and the distance between GBS *r* and vehicle *v* be denoted as $d_{r,v}^{t}$, they can be modeled as:

(7) $$d_{u,v}^{t}=\sqrt{{\left\|CU_{u}^{t}-CO_{v}^{t}\right\|}^{2}+{\left(H_{u}\right)}^{2}},d_{r,v}^{t}=\left\|CO_{r}-CO_{v}^{t}\right\|$$

where fixed constant $H_{u}$ denotes the flight altitude of UAV *u*, and the trajectory optimization is performed on the horizontal plane. This formulation is widely adopted in UAV trajectory optimization literature [17,18,19,20,21], because it reduces optimization dimensionality while maintaining the key geometric relationships between UAVs and ground vehicles. The chosen value of $H_{u}$ is specified later in Section 6. Although more complex channel effects—such as shadowing, Doppler variations, and altitude-dependent fading—are not included in the current model, they can be incorporated in future extensions without affecting the validity of the proposed optimization framework.

### 3.1. Communication Resource Allocation Model

Let the variable $cb\in \left\{cb_{v,r}^{t},cb_{v,u}^{t}\right\}$ represent the vehicle association decisions, where $cb_{v,r}^{t}\in \left\{0,1\right\}$, $cb_{v,u}^{t}\in \left\{0,1\right\}$. Let $cb_{v,r}^{t}=1$ if vehicle *v* is associated with GBS *r* at time slot *t*, and 0 otherwise. Similarly, let $cb_{v,u}^{t}=1$ if vehicle *v* is associated with UAV *u* at time slot *t*, and 0 otherwise. Since a vehicle can only be associated with one GBS or UAV at the same time slot, there are the following constraints:

(8) $$\sum_{r=1}^{R}cb_{r,v}^{t}+\sum_{u=1}^{U}cb_{u,v}^{t}=1,\forall v\in VE,t\in TI$$

Assume that the available spectrum in the network is divided into *C* orthogonal subchannels, which are represented by the set CN={1,2,…,C}, and all these subchannels have uniform bandwidth $B_{o}$. These subchannels are allocated to vehicles to support downlink data communication and radar sensing from edge nodes to vehicles. Let $ca=\left\{ca_{v}^{t,c}\right\}$ represent the subchannel allocation decision, where $ca_{v}^{t,c}\in \left\{0,1\right\}$. let $ca_{v}^{t,c}=1$ if subchannel *c* is allocated to vehicle *v* at time slot *t*, and 0 otherwise. To avoid co-channel interference, a single subchannel can only be allocated to a single vehicle. Therefore, the subchannel allocation decision is subject to the following constraints:

(9) $$\sum_{v=1}^{VE}ca_{v}^{t,c}=1,\forall c\in CN,t\in TI$$

In this paper, the wireless channel is modeled using a distance-dependent large-scale path loss expression. This simplified model follows related UAV-assisted vehicular communication works [17,18,19], where LoS propagation dominates in open-road or highway scenarios with limited vertical obstructions. The model effectively captures the dominant propagation characteristics of low-altitude UAV–vehicle links while allowing tractable analysis of ISAC performance. The free space path loss model is employed to characterize the channel gain between the UAV and the vehicle. The channel gain between UAV *u* and vehicle *v* on subchannel *c* can be expressed as follows:

(10) $$h_{u,v}^{t,c}=\frac{h_{u,v}^{c}}{{\left(d_{u,v}^{t}\right)}^{2}}$$

where $d_{u,v}^{t}$ denotes the Euclidean distance between UAV *u* and vehicle *v*, and $h_{v,u}^{c}$ represents the channel gain between UAV *u* and vehicle *v* at a reference distance of 1 m on subchannel *c*. The data transmission rate between UAV *u* and vehicle *v* on subchannel *c* can be expressed as:

(11) $$R_{u,v}^{t,c}=ca_{v}^{t,c}\cdot sccr^{t,c}\cdot B_{o}\cdot \log_{2}\left(1+\frac{h_{u,v}^{t,c}\cdot P_{u}^{t}}{N_{0}}\right)$$

where $N_{0}$ denotes the power of Gaussian white noise, $P_{u}^{t}$ represents the transmit power of UAV *u*, and the variable $sccr^{t,c}\in \left\{0,1\right\}$ represents the subchannel function selection decision. $sccr^{t,c}=1$ if subchannel *c* is allocated for data communication from the edge node to the vehicle at time slot *t*, and $sccr^{t,c}=0$ if subchannel *c* is allocated for radar sensing from the edge node to the vehicle at time slot *t*. Following subchannel allocation, the total achievable data transmission rate from UAV *u* to vehicle *v* can be represented as:

(12) $$TR_{u,v}^{t}=\sum_{c=1}^{C}R_{u,v}^{t,c}$$

The data transmission rate between GBS *r* and vehicle *v* on subchannel *c* can be expressed as:

(13) $$R_{r,v}^{t,c}=ca_{v}^{t,c}\cdot sccr^{t,c}\cdot B_{o}\cdot \log_{2}\left(1+\frac{P_{r}^{t}\cdot h_{r,v}^{t,c}}{N_{0}}\right)$$

where $h_{r,v}^{t,c}=h_{r,v}^{c}/{\left(d_{r,v}^{t}\right)}^{a}$ denotes the channel gain between GBS *r* and vehicle *v*, a≥0 represents the path loss exponent, $h_{r,v}^{c}$ represents the channel gain between GBS *r* and vehicle *v* at a reference distance of 1 m on subchannel *c*, and $P_{r}^{t}$ represents the transmit power of GBS *r*. Similarly, after subchannel allocation, the total achievable data transmission rate between GBS *r* and vehicle *v* can be expressed as:

(14) $$TR_{r,v}^{t}=\sum_{c=1}^{C}R_{r,v}^{t,c}$$

Considering the data transmission rates in both the vehicle-to-UAV and vehicle-to-GBS communication modes, the achievable data rate for vehicle *v* can be expressed as:

(15) $$TR_{v}^{t}=\sum_{r=1}^{R}\left(cb_{r,v}^{t}\cdot TR_{r,v}^{t}\right)+\sum_{u=1}^{U}\left(cb_{u,v}^{t}\cdot TR_{u,v}^{t}\right)$$

To meet the basic data rate requirements of vehicles, the achievable data rate for vehicle *v* is subject to the following constraint:

(16) $$TR_{v}^{t}\geq TR_{v}^{req},\forall v\in VE,t\in TI$$

where $TR_{v}^{req}$ denotes the minimum achievable communication rate threshold for vehicle *v*. Based on the above mathematical model, the total communication rate for all vehicles can be expressed as:

(17) $$TR^{t}=\sum_{v=1}^{V}TR_{v}^{t}$$

### 3.2. Radar Sensing Resource Allocation Model

The OFDM signals with sensing capabilities emitted by the dual-function transmitter from UAVs or GBSs will be reflected back to the UAVs or GBSs for decoding and processing after encountering the sensed vehicles. When subchannel c is allocated for radar sensing of vehicle *v*, the radar sensing performance, i.e., the MI between the target impulse response and the received signal, can be expressed as:

(18) $$MI_{r,v}^{t,c}=I\left(z_{r,v}^{t,c};q_{r,v}^{t,c}|s_{r,v}^{t,c}\right)=\frac{1}{2}\cdot B_{o}\cdot S\cdot T_{s}\cdot \log_{2}\left(1+\frac{P_{r}^{t}\cdot S\cdot T_{s}^{2}\cdot |Q_{r,v}^{c}(f^{c}){|}^{2}}{\sigma_{r,rad}^{c}}\right)$$

(19) $$MI_{u,v}^{t,c}=I\left(z_{u,v}^{t,c};q_{u,v}^{t,c}|s_{u,v}^{t,c}\right)=\frac{1}{2}\cdot B_{o}\cdot S\cdot T_{s}\cdot \log_{2}\left(1+\frac{P_{u}^{t}\cdot S\cdot T_{s}^{2}\cdot |Q_{u,v}^{c}(f^{c}){|}^{2}}{\sigma_{u,rad}^{c}}\right)$$

where $z_{u,v}^{t,c}$ and $z_{r,v}^{t,c}$ represent the reflected signals received by UAV *u* and GBS *r* on subchannel *c* regarding vehicle *v*. $q_{u,v}^{t,c}$ and $q_{r,v}^{t,c}$ represent the target impulse responses obtained by UAV *u* and GBS *r* when sending radar signals to vehicle *v* on subchannel *c*, and they are assumed to be Gaussian random processes [29]. $s_{u,v}^{t,c}$ and $s_{r,v}^{t,c}$ represent the waveforms sent by UAV *u* and GBS *r* to vehicle *v* on subchannel *c*, which consist of S consecutive OFDM symbols. $T_{s}$ is the duration of an OFDM symbol with a cyclic prefix, i.e., the time required to complete an OFDM symbol. $P_{u}^{t}$ and $P_{r}^{t}$ represent the transmit powers of UAV *u* and GBS *r*, respectively. $\sigma_{u,rad}^{c}$ and $\sigma_{r,rad}^{c}$ represent the noise powers at the radar receivers of UAV *u* and GBS *r* on the *c*-th subcarrier, respectively. $|Q_{r,v}^{c}(f^{c}){|}^{2}$ and $|Q_{u,v}^{c}(f^{c}){|}^{2}$ represent the squared magnitudes of the Fourier transforms of $q_{r,v}^{t,c}$ and $q_{u,v}^{t,c}$, respectively.

Based on the above mathematical model, the total MI obtained by UAV *u* for radar sensing of vehicle *v* can be expressed as:

(20) $$TMI_{u,v}^{t}=\sum_{c=1}^{C}\left(ca_{v}^{t,c}\cdot \left(1-sccr^{t,c}\right)\cdot MI_{u,v}^{t,c}\right)$$

The total MI obtained by GBS *r* for radar sensing of vehicle *v* can be expressed as:

(21) $$TMI_{r,v}^{t}=\sum_{c=1}^{C}\left(ca_{v}^{t,c}\cdot \left(1-sccr^{t,c}\right)\cdot MI_{r,v}^{t,c}\right)$$

The achievable MI obtained by the edge node for radar sensing of vehicle *v* can be represented as:

(22) $$TMI_{v}^{t}=\sum_{r=1}^{R}\left(cb_{v,r}^{t}\cdot TMI_{r,v}^{t}\right)+\sum_{u=1}^{U}\left(cb_{v,u}^{t}\cdot TMI_{u,v}^{t}\right)$$

To meet the basic requirements of radar sensing performance, the achievable MI obtained by the edge node for radar sensing of vehicle *v* is subject to the following constraint:

(23) $$TMI_{v}^{t}\geq HMI,\forall v\in VE,t\in TI$$

where HMI denotes the minimum MI requirement threshold for vehicle *v*.

Although MI is adopted as the sensing-performance indicator in this work, it is theoretically connected to classical radar-detection metrics. Under the Gaussian echo model, MI is monotonically related to the Kullback–Leibler divergence (KLD) between the target-present and target-absent hypotheses, where KLD directly determines the optimal detection probability *Pd* and false-alarm probability *Pfa* under the Neyman–Pearson detection framework [30,31]. Moreover, higher MI corresponds to reduced parameter-estimation uncertainty, leading to a tighter Cramer–Rao Bound (CRB) in radar parameter estimation [32]. Therefore, MI serves as a unified surrogate for *Pd*, *Pfa*, and CRB in ISAC sensing optimization. While MI is used in this study due to its analytical tractability and suitability for joint optimization, the proposed framework can be extended to explicitly incorporate detection-oriented metrics such as *Pd*, *Pfa* or CRB in future work.

## 4. Problem Formulation

In this paper, we propose the TDRA strategy to address the issue of decreased efficiency in communication and sensing service provision caused by the mutual constraints between communication and sensing functions, as well as the irrational planning of UAV flight trajectories in the considered architecture. The strategy maximizes the average achievable rate within the constraints of data transmission rate, radar sensing performance, and UAV energy consumption by jointly optimizing vehicle association, subchannel allocation, subchannel function selection, and UAV trajectory planning decisions. The optimization problem corresponding to the proposed strategy is expressed as:

(24) $$P1:\;\begin{array}{cc} \max & f\left(s\right) \end{array}=\frac{1}{T}\cdot \sum_{t=1}^{T}TR^{t}$$

$$\mathrm{st}.\left(1\right),\;\left(2\right),\;\left(5\right),\;\left(6\right),\;\left(8\right),\;\left(9\right),\;\left(16\right),\;\left(23\right)$$

(25) $$x_{u}^{t}\in \left[0,x_{\max}\right],y_{u}^{t}\in \left[0,y_{\max}\right],\forall t\in TI,u\in UA$$

(26) $$cb_{v,r}^{t}\in \left\{0,1\right\},cb_{v,u}^{t}\in \left\{0,1\right\},\forall v\in VE,r\in RU,u\in UA,t\in TI$$

(27) $$ca_{v}^{t,c}\in \left\{0,1\right\},\forall v\in VE,t\in TI,c\in CN$$

(28) $$sccr^{t,c}\in \left\{0,1\right\},\forall t\in TI,c\in CN$$

where $s=\left\{ca,sccr,CU,cb\right\}$ denotes the set of decision variables, $ca=\left\{ca_{v}^{t,c}\right\}$ represents the subchannel allocation decision, $sccr=\left\{sccr^{t,c}\right\}$ represents the subchannel function selection decision, $CU=\left\{CU_{u}^{t}\right\}$ represents the UAV trajectory decision, and $cb\in \left\{cb_{v,r}^{t},cb_{v,u}^{t}\right\}$ represent the vehicle association decision. Constraint (1) represents the UAV’s flight speed constraint, constraint (2) defines the beginning and ending points of the UAV’s flight, constraint (5) represents UAV’s energy consumption constraint, and constraint (6) ensures that the flight distance between UAVs remains within a safe interval. Constraint (8) indicates that a vehicle can only be associated with one edge node in a single time slot. Constraint (9) indicates that a single subchannel can only be allocated to one vehicle. Constraint (16) represents the communication rate performance constraint for vehicles. Constraint (23) represents the radar sensing performance constraint. Constraint (25) specifies the range of values for coordinates of the UAV’s position. Constraint (26) is boundary constraint for the vehicle association decision. Constraint (27) represents the boundary constraint for the subchannel allocation decision. Constraint (28) represents the boundary constraint for the subchannel function selection decision. 

In the optimization problem P1, the integer variables-including the vehicle association cb, the subchannel allocation ca, and the subchannel function selection sccr are multiplicatively coupled with the continuous UAV trajectory decision CU in both the objective function and the constraints (16) and (23). For example, the communication rate expression in (11), which forms the objective and constraint (16), contains products such as $ca_{v}^{t,c}\cdot sccr^{t,c}\cdot B_{o}\cdot \log_{2}\left(1+\left(h_{u,v}^{t,c}\cdot P_{u}^{t}\right)/N_{0}\right)$, where the discrete decision variables ca and sccr are multiplied with the logarithmic function of the channel gain $h_{u,v}^{t,c}\left(CU\right)$ and the channel gain itself is a nonlinear function of the UAV trajectory decision CU. Similarly, the sensing MI expression in (22), which forms constraint (23), contains multiplicative couplings among the integer variables cb, ca, and sccr. These nonlinear multiplicative coupling terms between discrete and continuous variables make P1 a typical mixed-integer nonlinear programming (MINLP) problem, which is difficult to solve to global optimality in polynomial time.

Block coordinate descent [33] is an iterative optimization method that updates only one or a few variables at each iteration while keeping the other variables fixed. This algorithm is particularly suitable for optimization problems with a large number of variables where the dependence of each variable on the others is relatively weak. In each iteration, the BCD method fixes all variables except one block and updates the variables in that block to maximize or minimize the objective function with respect to the current block. In this way, the block coordinate descent method gradually approaches the global or local optimal solution. The advantage of the block coordinate descent method is its high computational efficiency, especially when solving large-scale optimization problems. Compared with traditional optimization methods that require updating all variables simultaneously, the block coordinate descent method can significantly reduce the computational load of each iteration.

The decomposition of problem P1 into the three sub-problems follows BCD framework. Although global optimality cannot be guaranteed for non-convex MINLP problems, the decomposition preserves block-wise optimality because each sub-problem is solved exactly with respect to its own block of variables while the other blocks are held fixed. Consequently, each iteration yields an objective value no worse than that of the previous iteration, ensuring a monotonically non-decreasing sequence of objective values. It is worth emphasizing that each sub-problem is an exact reformulation of P1 with respect to its associated block variables, and therefore no relaxation gap is introduced across blocks. The approximation arises solely from the first-order SCA convexification, which constructs a tight lower-bound surrogate for the non-convex terms at each iteration. This ensures that every sub-problem solution remains feasible to the original problem and that the feasible region is preserved across iterations.

Based on classical convergence results of BCD methods for non-convex optimization [34], the monotonic improvement together with block-wise optimality implies that the proposed BCD–SCA iterative procedure converges to a stationary point of the original MINLP. Thus, although the solution is not globally optimal, it is theoretically meaningful, stable, and consistent with the commonly accepted notion of approximate optimality for large-scale MINLP problems.

## 5. Proposed Algorithm

### 5.1. Vehicle Association Subproblem

By fixing the subchannel allocation decision variables ca, the subchannel function selection decision variables sccr, and the UAV trajectory decision variables CU, and removing the constraints and terms in the objective function of optimization problem P1 that are unrelated to the vehicle association decision variables cb, we can obtain the vehicle association subproblem:

(29) $$P1.1:\;\max\;\;\;f\left(cb\right)=\frac{1}{T}\cdot \sum_{t=1}^{T}\sum_{v=1}^{V}\left(\sum_{r=1}^{R}\left(cb_{r,v}^{t}\cdot TR_{r,v}^{t}\right)+\sum_{u=1}^{U}\left(cb_{u,v}^{t}\cdot TR_{u,v}^{t}\right)\right)$$

$$st.\left(8\right)\;,\left(16\right)\;,\left(23\right)\;,\left(26\right)$$

The objective function of optimization problem P1.1 is a linear function, constraint (8) is an equality linear constraint, constraints (16) and (23) are inequality linear constraints, and the decision variables cb are 0–1 integer variables. Therefore, optimization problem P1.1 is a constrained integer linear programming problem. To facilitate solving, the variables cb are relaxed to continuous variables using linear relaxation techniques [35], i.e., $cb_{v,r}^{t}\in \left\{0,1\right\}\Rightarrow cb_{v,r}^{t}\in \left[0,1\right]$ and $cb_{v,u}^{t}\in \left\{0,1\right\}\Rightarrow cb_{v,u}^{t}\in \left[0,1\right]$, transforming optimization problem P1.1 into a linear programming problem with equality and inequality constraints.

The interior-point method is widely used to address linear programming problems [36]. It is a method based on duality theory and nonlinear optimization techniques. By searching within the interior of the feasible region, it avoids the unnecessary computations associated with searching along the boundary of the feasible region as in the simplex method [37]. The advantage of the interior-point method is its fast convergence speed, making it suitable for large-scale linear programming and convex optimization problems. It can also handle cases with unbounded or degenerate solutions. Hence, the interior-point method is employed to solve linear programming and convex optimization problems. 

The interior-point method transforms constrained optimization problems into unconstrained ones by introducing barrier functions and then solves them using unconstrained optimization algorithms such as Newton’s method. The convex optimization problem with equality constraints obtained by transforming problem P1.1 using the logarithmic barrier method can be formulated as:

(30) $$P1.1.1:\;\min\;\;\;F(cb)=f\left(cb\right)+\mu F1C(cb)$$

$$st.\left(8\right)$$

where μ>0 is the penalty parameter, and F1C(cb) is the logarithmic barrier function, which is expressed as:

(31) $$\begin{array}{l} F1C(cb)=-\sum_{t=1}^{T}\sum_{v=1}^{V}\log\left(TR_{v}^{t}-TR_{v}^{req}\right)-\sum_{t=1}^{T}\sum_{v=1}^{V}\log\left(TMI_{v}^{t}-HMI_{v}^{t}\right)\\ -\sum_{t=1}^{T}\sum_{v=1}^{V}\sum_{r=1}^{R}\log\left(1-cb_{v,r}^{t}\right)-\sum_{t=1}^{T}\sum_{v=1}^{V}\sum_{r=1}^{R}\log cb_{v,r}^{t}\\ -\sum_{t=1}^{T}\sum_{v=1}^{V}\sum_{u=1}^{U}\log\left(1-cb_{v,u}^{t}\right)-\sum_{t=1}^{T}\sum_{v=1}^{V}\sum_{u=1}^{U}\log cb_{v,u}^{t} \end{array}$$

Since optimization problem P1.1.1 still has equality constraint (8), it cannot be directly solved using methods for unconstrained problems such as Newton’s method. Therefore, the exterior penalty function method [33] is used to transform equality constraint (8) into an exterior penalty function in the objective function, thus converting optimization problem P1.1.1 into an optimization problem with a mixed augmented objective function that includes both exterior penalty functions and logarithmic barrier functions:

(32) $$P1.1.2:\;\min\;\;\;F(cb)=f\left(cb\right)+\mu F1C(cb)+\frac{1}{2\mu }\sum_{t=1}^{T}\sum_{v=1}^{V}{\left(g_{v}^{t}\left(cb\right)\right)}^{2}$$

where $g_{v}^{t}\left(cb\right)=\left(\sum_{r=1}^{R}cb_{r,v}^{t}+\sum_{u=1}^{U}cb_{u,v}^{t}\right)-1$. Optimization problem P1.1.2 is an unconstrained convex optimization problem, which can be solved using Newton’s method. For the optimal relaxed solution $cb^{*}=\left\{c{b_{r,v}^{t}}^{*},c{b_{u,v}^{t}}^{*}\right\}$ obtained by solving optimization problem P1.1.2, this paper uses the random rounding method [38] to convert the relaxed solution to an integer solution. The mathematical model of the random rounding method can be denoted as follows:

(33) $$R\left[\Psi =1\right]=\Psi^{∼}$$

where $\Psi^{∼}$ denotes the relaxed solution of the optimization problem, and $R\left[\Psi =1\right]$ represents the probability that the 0–1 variable Ψ is 1.

The vehicle association algorithm is presented in Algorithm 1, and the procedure is as follows: (1) Initializing all types of values and setting the algorithm termination iteration threshold. (2) Relaxing constraint (26) to transform P1.1 into a constrained linear programming problem. (3) Using the logarithmic barrier method to transform P1.1 into a convex programming problem P1.1.1 with equality constraints. (4) Using the exterior penalty function method to transform P1.1.1 into an unconstrained convex problem P1.1.2. (5) In each iteration, Newton’s method is used to solve P1.1.2, and the initial solution $cb^{*∼}$ and the penalty parameter μ are updated to serve as the initial solution and penalty parameter for the next iteration. This process continues until the termination condition is met. (6) After the iteration ends, the random rounding method is employed to convert the optimal relaxed solution into an optimal vehicle association scheme.
**Algorithm 1:** Vehicle Association Algorithm**Input:** Initial solution $cb^{*∼}\leftarrow cb^{0}$, penalty parameter $\mu \leftarrow \mu^{0}>0$, decrement coefficient $\lambda \in \left(0,1\right)$, error threshold χ, algorithm iteration count l←0, maximum algorithm iteration count L>0. **Output:** Approximate optimal solution for vehicle association $cb^{*}$**1:**The constraint (26) is relaxed to transform the optimization problem P1.1 into a constrained linear programming problem. **2:**The logarithmic barrier method is used to transform P1.1 into a convex problem P1.1.1 with equality constraints; **3:**The exterior penalty function method is used to transform P1.1.1 into an unconstrained convex problem P1.1.2; **4:****repeat****5:**l←l+1;**6:**Based on the initial solution $cb^{*∼}$ and the penalty parameter μ, Newton’s method is used to solve P1.1.2 to obtain the solution $cb^{l}$ for the current iteration; **7:**Updating the initial solution **8:**Updating the penalty parameter **9:****until** $\left|cb^{l}-cb^{l-1}\right|\leq \chi$ or l≥L**10:**$cb^{*}\leftarrow R\left[cb=1\right]=cb^{l}$;**11****return** $cb^{*}$

### 5.2. UAV Trajectory Planning Subproblem

By fixing the subchannel allocation decision variables ca the subchannel function selection decision variables sccr, and the vehicle association decision variables, cb and by removing the constraints and corresponding terms in the objective function of optimization problem P1 that are unrelated to the UAV trajectory decision variables CU, we can obtain the UAV trajectory planning subproblem:

(34) $$P1.2:\;\max\;\;\;f\left(CU\right)=\frac{1}{T}\cdot \sum_{t=1}^{T}\sum_{v=1}^{V}\sum_{u=1}^{U}\sum_{c=1}^{C}\left(\begin{array}{l} cb_{u,v}^{t}\cdot ca_{v}^{t,c}\cdot sccr^{t,c}\cdot B_{o}\\ \cdot \log_{2}\left(1+\frac{h_{u,v}^{c}\cdot P_{u}^{t}\cdot N_{0}^{-1}}{{\left\|CU_{u}^{t}-CO_{v}^{t}\right\|}^{2}+{\left(H_{u}\right)}^{2}}\right) \end{array}\right)$$

$$st.\left(1\right),\left(2\right),\left(5\right),\left(6\right),\left(16\right),\left(25\right)$$

The objective function of P1.2 is a non-convex function, and constraints (6) and (16) are non-convex constraints. This renders P1.2 a non-convex optimization problem, which is challenging to solve directly. The SCA method is employed to transform the non-convex problem into an approximately convex one, which is then solved using convex optimization techniques to obtain an approximate optimal solution.

Following the method in reference [36], we first introduce an auxiliary variable $g=\left\{g_{v}^{t}\right\}$ as the lower bound of the data transmission rate from the UAV to vehicle *v*, and transform the optimization problem into:

(35) $$P1.2.1:\;\max\;\;\;f\left(CU,g\right)=\frac{1}{T}\cdot \sum_{t=1}^{T}\sum_{v=1}^{V}g_{v}^{t}$$

$$st.\left(1\right),\;\left(2\right),\;\left(5\right),\;\left(6\right),\;\left(25\right)$$

(36) $$\left(\sum_{r=1}^{R}\left(cb_{r,v}^{t}\cdot TR_{r,v}^{t}\right)+g_{v}^{t}\right)\geq TR_{v}^{req},\forall v\in VE,t\in TI$$

(37) $$\begin{array}{l} \sum_{u=1}^{U}\sum_{c=1}^{C}\left(cb_{u,v}^{t}\cdot ca_{v}^{t,c}\cdot sccr^{t,c}\cdot B_{o}\cdot \log_{2}\left(1+\frac{h_{u,v}^{c}\cdot P_{u}^{t}\cdot N_{0}^{-1}}{{\left\|CU_{u}^{t}-CO_{v}^{t}\right\|}^{2}+{\left(H_{u}\right)}^{2}}\right)\right)\geq g_{v}^{t}\\ ,\forall v\in VE,t\in TI \end{array}$$

In optimization problem P1.2.1, the objective function is a linear function, constraint (36) is a linear constraint, and constraint (37) is still a non-convex constraint. The expression (38) contained in constraint (37) is a convex function of ${\left\|CU_{u}^{t}-CO_{v}^{t}\right\|}^{2}$, (38) can be expressed as:

(38) $$\delta_{v,u}^{t,c}=\log_{2}\left(1+\frac{h_{u,v}^{c}\cdot P_{u}^{t}\cdot N_{0}^{-1}}{{\left\|CU_{u}^{t}-CO_{v}^{t}\right\|}^{2}+{\left(H_{u}\right)}^{2}}\right)$$

The expression (38) can be approximated by its first-order Taylor expansion at any point ${\left\|{\bar{CU_{u}^{t}}}^{k}-CO_{v}^{t}\right\|}^{2}$ in the *k*-th iteration to obtain a global lower bound:

(39) $$\bar{\delta_{v,u}^{t,c}}={\bar{\delta_{v,u}^{t,c}}}^{k}+{\bar{\nabla \delta_{v,u}^{t,c}}}^{k}\left({\left\|CU_{u}^{t}-CO_{v}^{t}\right\|}^{2}-{\left\|{\bar{CU_{u}^{t}}}^{k}-CO_{v}^{t}\right\|}^{2}\right)$$

where ${\bar{\delta_{v,u}^{t,c}}}^{k}$ denotes the value of the function at point ${\left\|{\bar{CU_{u}^{t}}}^{k}-CO_{v}^{t}\right\|}^{2}$ in the *k*-th iteration, and ${\bar{\nabla \delta_{v,u}^{t,c}}}^{k}$ denotes the first-order derivative of the function $\delta_{v,u}^{t,c}$ at point ${\left\|{\bar{CU_{u}^{t}}}^{k}-CO_{v}^{t}\right\|}^{2}$ in the *k*-th iteration. Therefore, constraint (37) can be transformed into the following convex constraint:

(40) $$\sum_{u=1}^{U}\sum_{c=1}^{C}\left(cb_{u,v}^{t}\cdot ca_{v}^{t,c}\cdot sccr^{t,c}\cdot B_{o}\cdot \bar{\delta_{v,u}^{t,c}}\right)\geq g_{v}^{t},\forall v\in VE,t\in TI$$

The left-hand side expression of constraint (6) is a convex function with respect to variables $CU_{u}^{t}$ and $CU_{u^{\prime }}^{t}$. Therefore, the left-hand side expression of constraint (6) can be approximated by its first-order Taylor expansion at any points ${\bar{CU_{u}^{t}}}^{k}$ and ${\bar{CU_{u^{\prime }}^{t}}}^{k}$ to obtain a lower bound:

(41) $$\begin{array}{l} {\left\|CU_{u}^{t}-CU_{u^{\prime }}^{t}\right\|}^{2}\geq \chi ={\left\|{\bar{CU_{u}^{t}}}^{k}-{\bar{CU_{u^{\prime }}^{t}}}^{k}\right\|}^{2}+2{\left({\bar{CU_{u}^{t}}}^{k}-{\bar{CU_{u^{\prime }}^{t}}}^{k}\right)}^{T}\cdot \left(CU_{u}^{t}-{\bar{CU_{u}^{t}}}^{k}\right)\\ -2{\left({\bar{CU_{u}^{t}}}^{k}-{\bar{CU_{u^{\prime }}^{t}}}^{k}\right)}^{T}\cdot \left(CU_{u^{\prime }}^{t}-{\bar{CU_{u^{\prime }}^{t}}}^{k}\right) \end{array}$$

Based on (41), constraint (6) can be transformed into the following linear constraint:

(42) $$\chi \geq d_{\min}^{2},\forall u\in UA,u^{\prime }\in UA,u\neq u^{\prime },t\in TI$$

Therefore, the optimization problem P1.2.1 can be transformed into:

(43) $$P1.2.2:\;\max\;\;\;f\left(CU,g\right)$$

$$st.\left(1\right),\;\left(2\right),\;\left(5\right),\;\left(25\right),\;\left(36\right),\;\left(40\right),\;\left(42\right)$$

The optimization problem P1.2.2 is a convex optimization problem with constraints, solvable by the interior-point method.

To justify the validity of the first-order Taylor approximation used in the SCA procedure, we provide an upper bound on the approximation error. Let $f\left(x\right)$ be a convex and continuously differentiable function. Its first-order Taylor approximation at point $x^{k}$ is given by $\tilde{f}\left(x;x^{k}\right)=f\left(x^{k}\right)+\nabla f{\left(x^{k}\right)}^{T}\left(x-x^{k}\right)$. The Taylor remainder term satisfies the standard inequality for smooth convex functions $0\leq f\left(x\right)-\tilde{f}\left(x;x^{k}\right)\leq \left(L/2\right){\left\|x-x^{k}\right\|}^{2}$, where *L* is the Lipschitz constant of $\nabla f\left(x\right)$. Thus, the approximation error is quadratically bounded $\varepsilon \left(x\right)=f\left(x\right)-\tilde{f}\left(x;x^{k}\right)\leq \frac{L}{2}{\left\|x-x^{k}\right\|}^{2}$. Because SCA typically generates updates within a local neighborhood of the previous iterate, the squared term ensures that the approximation error becomes negligible near convergence. Moreover, the SCA method guarantees that $\tilde{f}\left(x;x^{k}\right)$ is a global lower bound of the original non-convex expression. Therefore, each iteration improves the objective function of the original problem, which ensures monotonic convergence toward a stationary point.

### 5.3. Subchannel Allocation Subproblem

By fixing the UAV trajectory decision variables CU and the vehicle association decision variables cb, and removing the constraints and corresponding terms in the objective function of optimization problem P1 that are unrelated to the subchannel allocation and subchannel function selection decisions sccr, we can obtain the subchannel allocation subproblem:

(44) $$P1.3:\;\max\;\;\;f\left(ca,sccr\right)=\frac{1}{T}\cdot \sum_{t=1}^{T}\sum_{v=1}^{V}\left(\begin{array}{l} \sum_{r=1}^{R}\sum_{c=1}^{C}\left(cb_{r,v}^{t}\cdot ca_{v}^{t,c}\cdot sccr^{t,c}\cdot B_{o}\cdot \log_{2}(1+\frac{P_{r}^{t}\cdot h_{r,v}^{t,c}}{N_{0}})\right)\\ +\sum_{u=1}^{U}\sum_{c=1}^{C}\left(cb_{u,v}^{t}\cdot ca_{v}^{t,c}\cdot sccr^{t,c}\cdot B_{o}\cdot \log_{2}(1+\frac{h_{u,v}^{t,c}\cdot P_{u}^{t}}{N_{0}})\right) \end{array}\right)$$

$$st.\left(9\right),\;\left(16\right),\;\left(23\right),\;\left(27\right),\;\left(28\right)$$

The objective function and constraints (16) and (23) of optimization problem P1.3 contain non-convex terms involving variable multiplication, making it difficult to solve. Therefore, an auxiliary variable $\varepsilon =\left\{\varepsilon_{v}^{t,c}\right\}$ is introduced to represent the lower bound of $ca_{v}^{t,c}\cdot sccr^{t,c}$. By replacing the relevant terms with ε, we can transform P1.3 into:

(45) $$P1.3.1:\;\max\;\;\;f\left(ca,sccr,\varepsilon \right)=\frac{1}{T}\cdot \sum_{t=1}^{T}\sum_{v=1}^{V}\left(\begin{array}{l} \sum_{r=1}^{R}\sum_{c=1}^{C}\left(cb_{r,v}^{t}\cdot \varepsilon_{v}^{t,c}\cdot B_{o}\cdot \log_{2}(1+\frac{P_{r}^{t}\cdot h_{r,v}^{t,c}}{N_{0}})\right)\\ +\sum_{u=1}^{U}\sum_{c=1}^{C}\left(cb_{u,v}^{t}\cdot \varepsilon_{v}^{t,c}\cdot B_{o}\cdot \log_{2}(1+\frac{h_{u,v}^{t,c}\cdot P_{u}^{t}}{N_{0}})\right) \end{array}\right)$$

$$st.(9),\;\left(27\right),\;\left(28\right)$$

(46) $$\left(\begin{array}{l} \sum_{r=1}^{R}\sum_{c=1}^{C}\left(cb_{r,v}^{t}\cdot \varepsilon_{v}^{t,c}\cdot B_{o}\cdot \log_{2}\left(1+\frac{P_{r}^{t}\cdot h_{r,v}^{t,c}}{N_{0}}\right)\right)\\ +\sum_{u=1}^{U}\sum_{c=1}^{C}\left(cb_{u,v}^{t}\cdot \varepsilon_{v}^{t,c}\cdot B_{o}\cdot \log_{2}\left(1+\frac{h_{u,v}^{t,c}\cdot P_{u}^{t}}{N_{0}}\right)\right) \end{array}\right)\geq TR_{v}^{req},\forall v\in VE,t\in TI$$

(47) $$\left(\begin{array}{l} \sum_{r=1}^{R}\sum_{c=1}^{C}\left(cb_{v,r}^{t}\cdot \left(ca_{v}^{t,c}-\varepsilon_{v}^{t,c}\right)\cdot MI_{r,v}^{t,c}\right)\\ +\sum_{u=1}^{U}\sum_{c=1}^{C}\left(cb_{v,u}^{t}\cdot \left(ca_{v}^{t,c}-\varepsilon_{v}^{t,c}\right)\cdot MI_{u,v}^{t,c}\right) \end{array}\right)\geq HMI_{v}^{t},\forall v\in VE,t\in TI$$

(48) $$\varepsilon_{v}^{t,c}\leq ca_{v}^{t,c}\cdot sccr^{t,c},\forall v\in VE,t\in TI,c\in CN$$

The objective function of optimization problem P1.3.1 is a linear function, constraints (46) and (47) are linear constraints, and constraint (48) is still a non-convex constraint containing the variable multiplication term $ca_{v}^{t,c}\cdot sccr^{t,c}$. To transform constraint (48) into a convex constraint, $ca_{v}^{t,c}\cdot sccr^{t,c}$ is approximated as:

(49) $$\left(ca_{v}^{t,c}\cdot sccr^{t,c}\right)=\frac{{\left(ca_{v}^{t,c}+sccr^{t,c}\right)}^{2}-\left({\left(ca_{v}^{t,c}\right)}^{2}+{\left(sccr^{t,c}\right)}^{2}\right)}{2}\geq \left(\frac{1}{2}\left(CC1-CC2\right)\right)$$

where CC1 and CC2 represent the first-order Taylor expansions of the expressions ${\left(ca_{v}^{t,c}+sccr^{t,c}\right)}^{2}$ and $\left({\left(ca_{v}^{t,c}\right)}^{2}+{\left(sccr^{t,c}\right)}^{2}\right)$ at any points ${\bar{ca_{v}^{t,c}}}^{k}$ and ${\bar{sccr^{t,c}}}^{k}$ in the *k*-th iteration:

(50) $$CC1=\left(\begin{array}{l} {\left({\bar{ca_{v}^{t,c}}}^{k}+{\bar{sccr^{t,c}}}^{k}\right)}^{2}+2\left({\bar{ca_{v}^{t,c}}}^{k}+{\bar{sccr^{t,c}}}^{k}\right)\cdot \left(ca_{v}^{t,c}-{\bar{ca_{v}^{t,c}}}^{k}\right)\\ +2\left({\bar{ca_{v}^{t,c}}}^{k}+{\bar{sccr^{t,c}}}^{k}\right)\cdot \left(sccr^{t,c}-{\bar{sccr^{t,c}}}^{k}\right) \end{array}\right)$$

(51) $$CC2=\left(\begin{array}{l} {\left({\bar{ca_{v}^{t,c}}}^{k}\right)}^{2}+{\left({\bar{sccr^{t,c}}}^{k}\right)}^{2}+2{\bar{ca_{v}^{t,c}}}^{k}\cdot \left(ca_{v}^{t,c}-{\bar{ca_{v}^{t,c}}}^{k}\right)\\ +2{\bar{sccr^{t,c}}}^{k}\cdot \left(sccr^{t,c}-{\bar{sccr^{t,c}}}^{k}\right) \end{array}\right)$$

Based on the above derivation, constraint (48) can be transformed into the following linear constraint:

(52) $$\varepsilon_{v}^{t,c}\leq \left(\frac{1}{2}\left(CC1-CC2\right)\right),\forall v\in VE,t\in TI,c\in CN$$

Thus, the optimization problem P1.3.1 can be converted into:

(53) $$P1.3.2\;\;\;\max\;\;\;f\left(ca,sccr,\varepsilon \right)$$

$$st.\left(9\right),\;\left(27\right),\;\left(28\right),\;\left(46\right),\;\left(47\right),\;\left(52\right)$$

By observation, the objective function of optimization problem P1.3.2 is a linear function, all constraints are linear constraints, and the decision variables ca and sccr are 0–1 integer variables. Therefore, optimization problem P1.3.2 is a constrained integer linear programming problem. First, ca and sccr are relaxed to continuous variables, i.e., $ca_{v}^{t,c}\in \left\{0,1\right\}\Rightarrow ca_{v}^{t,c}\in \left[0,1\right]$, $sccr^{t,c}\in \left\{0,1\right\}\Rightarrow sccr^{t,c}\in \left[0,1\right]$. Then, the interior-point method is used to solve the optimization problem to obtain the optimal relaxed solution for subchannel allocation. Finally, the random rounding method is used to restore the obtained optimal relaxed solutions $ca^{*}$ and $sccr^{*}$.

### 5.4. Algorithm Implementation and Analysis

This paper proposes a TDRA Algorithm for solving optimization problem P1. The algorithm is developed using the block coordinate descent, which alternately addresses the subproblems to achieve an approximate optimal solution for the original problem. Algorithm 2 displays the pseudocode of the proposed algorithm. The process of the algorithm is as follows: (1) The initial values for the set of decision variables are provided, and the error tolerance threshold and the maximum number of iterations are set. (2) In each iteration, UAV trajectory planning subproblem, vehicle association subproblem, and subchannel allocation subproblem are solved in sequence to obtain the solution of optimization problem P1 for the current iteration. (3) the error value in the current iteration is computed. If the error value is less than the set error tolerance threshold or the number of iterations exceeds the maximum iteration threshold, the algorithm terminates, and the approximate optimal solution to optimization problem P1 is obtained.
**Algorithm 2**: TDRA Algorithm**Input:** Decision variable set initial value $s\left(0\right)\leftarrow \left\{CU_{0},cb_{0},ca_{0},sccr_{0}\right\}$, error tolerance threshold χ, number of iterations l=0, maximum number of iterations L>0**Output:** Approximate optimal solution $s^{*}$**1.****repeat****2.**l←l+1**;****3.**$s\left(l\right)\leftarrow CU_{l}^{*}$;// The subproblem P1.2 is solved to obtain the UAV trajectory planning decision.**4.**$s\left(l\right)\leftarrow cb_{l}^{*}$;// The subproblem P1.1 is solved to obtain the vehicle association decision.**5.**$s\left(l\right)\leftarrow \left\{ca_{l}^{*},sccr_{l}^{*}\right\}$;// The subproblem P1.3 is solved to obtain the subchannel allocation and function selection decisions.**6.****until** $\left|s\left(l\right)-s\left(l-1\right)\right|\leq \chi$ **or** l≥L**7.**$s^{*}\leftarrow s\left(l\right)$**;****8.****return** $s^{*}$

The time complexity of using the interior-point method to solve subproblem P1.1 is $O\left(tc_{1.1}=L_{1.1}\cdot {\left(TV\left(R+U\right)\right)}^{3.5}\right)$, where *R*, *U*, *V*, and *T* represent the number of GBSs, UAVs, vehicles, and time slots in the time period, respectively. $L_{1.1}$ is the iterations number required to solve subproblem P1.1, $TV\left(R+U\right)$ is the size of subproblem P1.1, and $O\left(TV\left(R+U\right)\right)$ is the complexity required for random rounding of the relaxed variable. The time complexity of solving subproblem P1.2 is $O\left(tc_{1.2}=L_{1.2}\cdot {\left(T\left(2U+V\right)\right)}^{3.5}\right)$, where $L_{1.2}$ is the iteration number required to solve subproblem P1.2, and $T\left(2U+V\right)$ is the size of subproblem P1.2. The time complexity of solving subproblem P1.3 is $O\left(tc_{1.3}=L_{1.3}\cdot {\left(TC\left(2V+1\right)\right)}^{3.5}\right)$, where *C* is the number of subchannels, $TC\left(2V+1\right)$ is the size of subproblem P1.3, $L_{1.3}$ is the iterations number required to solve the optimization problem, and $O\left(TC\left(V+1\right)\right)$ is the complexity required for random rounding. Therefore, the time complexity of Algorithm 2 can be expressed as $O\left(L_{1}\cdot \max\left(tc_{1.1},tc_{1.2},tc_{1.3}\right)\right)$, where $L_{2}$ is the iterations number of Algorithm 2.

The convergence analysis of Algorithm 2 is as follows: In the (*l* + 1)-th iteration, solving optimization problem P1.2 given the vehicle association decision $\left\{cb_{l}\right\}$ and the subchannel allocation decision $\left\{ca_{l},sccr_{l}\right\}$ can yield the approximate optimal solution for the UAV trajectory planning decision $\left\{CU_{l+1}\right\}$. At this point, there exists:

(54) $$f\left(CU_{l+1},cb_{l},ca_{l},sccr_{l}\right)\geq f\left(CU_{l},cb_{l},ca_{l},sccr_{l}\right)$$

Given the UAV trajectory planning decision $\left\{CU_{l}\right\}$ and the subchannel allocation decision $\left\{ca_{l},sccr_{l}\right\}$, solving optimization problem P2.1 can yield the approximate optimal solution for the vehicle association decision $\left\{cb_{l+1}\right\}$. Then, there exists:

(55) $$f\left(CU_{l},cb_{l+1},ca_{l},sccr_{l}\right)\geq f\left(CU_{l},cb_{l},ca_{l},sccr_{l}\right)$$

Similarly, given the UAV trajectory planning decision $\left\{CU_{l}\right\}$ and the vehicle association decision $\left\{cb_{l}\right\}$, solving optimization problem P1.3 can yield the approximate optimal solution for the subchannel allocation and subchannel function selection decisions. $\left\{ca_{l+1},sccr_{l+1}\right\}$ Then, there exists:

(56) $$f\left(CU_{l},cb_{l},ca_{l+1},sccr_{l+1}\right)\geq f\left(CU_{l},cb_{l},ca_{l},sccr_{l}\right)$$

In summary, in the (*l* + 1)-th iteration, solving the subproblems P1.2, P1.1, and P1.3 in sequence results in a solution that ensures the objective function value of P1 consistently exceeds or equals the objective function value at the *l*-th iteration., i.e.:

(57) $$f\left(ca_{l+1},sccr_{l+1},CU_{l+1},cb_{l+1}\right)\geq f\left(ca_{l},sccr_{l},CU_{l},cb_{l}\right)$$

Therefore, in each iteration, the objective function of P1 is non-decreasing. Given a precision threshold, a finite number of iterations can yield an upper bound on the objective function of optimization problem P1. It can be proven that Algorithm 2 is convergent.

These properties satisfy the standard Majorization Minimization (MM) conditions, ensuring monotonic non-decreasing objective values at each iteration. Therefore, the BCD–SCA procedure converges to a stationary point of the original problem according to established BCD convergence theory.

In practical UAV-assisted vehicular networks, the system is subject to external dynamic disturbances such as small-scale vehicle mobility, channel fluctuations, and UAV trajectory perturbations. The proposed BCD–SCA iterative algorithm inherently accommodates such disturbances due to the following reasons:

(1) Time-slot-based quasi-static modeling: Each time slot is sufficiently short (1 s) such that the channel and node positions can be approximated as quasi-static. This modeling approach is widely adopted in UAV–V2X literature and is robust to small disturbances within each time slot.

(2) Lower-bound approximation in SCA: The first-order Taylor approximation used in SCA forms a global lower bound of the original non-convex function. Therefore, even under mild environmental fluctuations, the optimization update still guarantees monotonic improvement of the objective function.

(3) Local robustness of BCD updates: At each iteration, BCD optimizes one block while fixing others. This structure ensures that small perturbations in trajectory or channel conditions only affect one block at a time, preventing error propagation across variables.

(4) Trajectory smoothing effect: The UAV trajectory is optimized over multiple time slots. Sudden disturbances in one time slot have limited impact on overall trajectory design because the optimization is performed over the entire planning horizon.

## 6. Simulation Results and Analysis

In this chapter, the numerical simulations are employed to perform experiments and evaluate the performance of the proposed algorithm. First, the experimental environment and parameter settings for the numerical simulation are introduced. Second, the comparison algorithms and performance evaluation metrics are described. Then, the simulation results and performance analysis of the algorithms are presented. Finally, we summarize the experimental conclusions.

### 6.1. Simulation Environment and Parameter Settings

**Figure 2:**
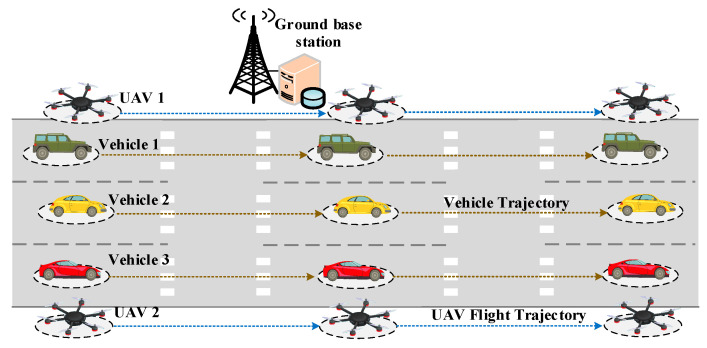
Illustration of simulation scenario diagram.

The numerical simulation experiments are run on an Alienware m17 R4 laptop with a performance configuration of 32 GB RAM and a 2.4 GHz Intel Core i9 processor. The software environment is Python 3.11. Figure 2 illustrates the simulation scenario diagram of the algorithm. The simulation scenario simulates a 150 m × 30 m section of a one-way three-lane highway in a temporary traffic congestion state, with GBSs randomly deployed along the roadside. Multiple vehicles drive on this highway at a speed of 2 m/s [39], with random starting positions. UAVs fly according to predetermined starting and ending positions, and their flight routes can be optimized through the algorithm. This moderate-sized environment enables detailed analysis of vehicle association, ISAC performance, and UAV trajectory behavior while avoiding excessive randomness associated with large-scale networks. Although the simulation area is limited, the proposed BCD–SCA optimization framework is not restricted by scenario size and can be readily extended to larger or more complex urban environments. Future work will also explore extended simulation scales, altitude variations, acceleration constraints, and urban-blockage-aware channel models.

To ensure safe low-altitude operation and realistic sensing and communication performance, the UAVs are deployed at a fixed altitude of 8 m. This design choice follows common practice in UAV sensing and UAV-assisted vehicular edge computing studies [40,41,42,43,44], where UAVs typically operate within 5–30 m above roadways to obtain high-resolution perception data and maintain reliable connectivity. Operating at such low altitudes improves LoS probability and reduces pathloss, thereby enhancing communication and sensing quality in temporary congestion scenarios. Furthermore, the selected altitude lies well within the Visual Line-of-Sight (VLOS) operation range specified in FAA Part 107 [45], which allows UAV flights below 120 m in controlled areas. Considering that vehicles and surrounding roadside structures are generally below 4–5 m, an 8 m altitude provides unobstructed coverage for both communication and sensing tasks. In addition, the minimum inter-UAV safety distance is set to 8 m, consistent with parameter settings in multi-UAV ISAC and vehicular networking studies [46,47,48,49], ensuring operational robustness under minor wind disturbances and trajectory corrections.

Since the proposed algorithm is applicable for optimization in a multi-time-slot scenario, the simulation scenario designed above is multi-time-slot. In the multi-time-slot simulation scenario, the mobility of dynamic nodes such as UAVs and vehicles are reflected by the changes in their positions across different time slots, while the positions of ground edge nodes remain stationary. In each time slot, it is assumed that these dynamic nodes are in a quasi-static state. Therefore, the multi-time-slot scenario considered characterizes the mobility of dynamic nodes through a continuous change in time slots. All relevant system parameters used in the simulations are summarized in Table 1.

### 6.2. Comparison Algorithms and Evaluation Metrics

This paper selects following comparison algorithms for evaluating the proposed algorithm, which include: 

(1) Algorithm with User association Random Assignment (URA) [50]: The algorithm maximizes the downlink rate by optimizing UAV trajectory planning and subchannel allocation. However, this algorithm does not consider optimizing the communication association decision between the user and the edge node. This paper assumes that this algorithm randomly assigns the communication association between users and edge nodes.

(2) Joint optimization algorithm with Subchannel allocation and User Association (SAUA) [51]: The algorithm maximizes the communication rate by jointly optimizing user association and subchannel allocation, but it does not consider optimizing the UAV trajectory. This paper assumes that the UAV in this algorithm flies using an unoptimized trajectory. 

(3) Algorithm with Sub-channel Equal Allocation (SEA) [20]: The algorithm maximizes the communication rate between users and edge nodes by jointly optimizing UAV trajectory planning and user association. The algorithm does not consider optimizing subchannel allocation. This paper assumes that the algorithm allocates subchannel resources evenly.

In addition, this paper selects the average achievable rate (24) as the performance evaluation metric for the algorithms.

### 6.3. Performance Comparison

This paper evaluates the convergence of the proposed algorithm, then analyzes the optimization results of UAV trajectory planning by the proposed algorithm. Subsequently, it investigates the impact of changes in influencing factors such as the number of subchannels, UAV transmission power, UAV maximum flight speed, minimum MI requirement, and subchannel bandwidth on algorithm performance. Each experiment set in this chapter is conducted 15 times, with the average of the tests taken as the final result.

**Figure 3:**
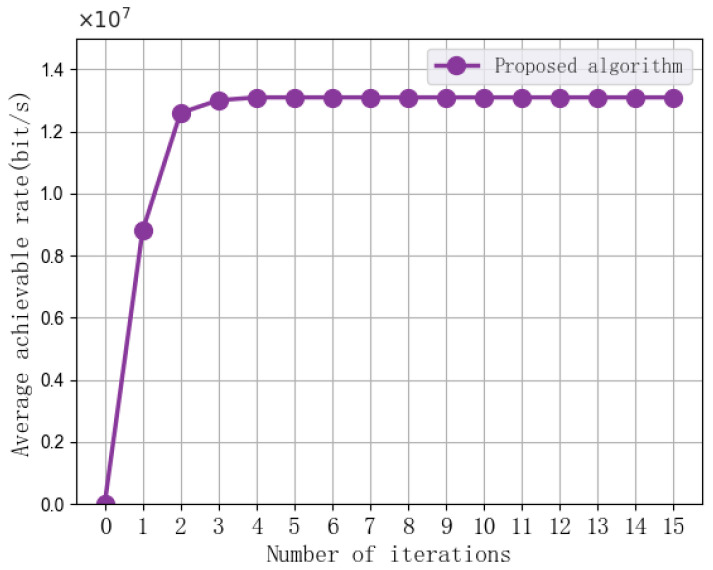
The convergence performance of the proposed algorithm.

Figure 3 shows the convergence results of the proposed algorithm. In this experiment, the algorithm iterates 15 times, with the UAV transmission power set at 2 W, the maximum UAV flight speed at 10 m/s, the subchannel bandwidth at 1 MHz, and the minimum MI requirement at 200 bits. As shown in the figure, the average achievable rate performance of the proposed algorithm gradually increases from the 1st to the 4th iteration, and then gradually converges from the 4th iteration onwards. This suggests that the proposed algorithm exhibits favorable convergence characteristics.

Furthermore, although the exact global optimum of the MINLP problem is not computable, the monotonic convergence curve shown in Figure 3 indicates that the approximation error introduced by the first-order Taylor expansion does not degrade the final performance. Because the SCA update ensures that the step size $\left\|x^{k+1}-x^{k}\right\|$ decreases as the algorithm converges, the error upper bound $\left(L/2\right){\left\|x-x^{k}\right\|}^{2}$ approaches zero.

**Figure 4:**
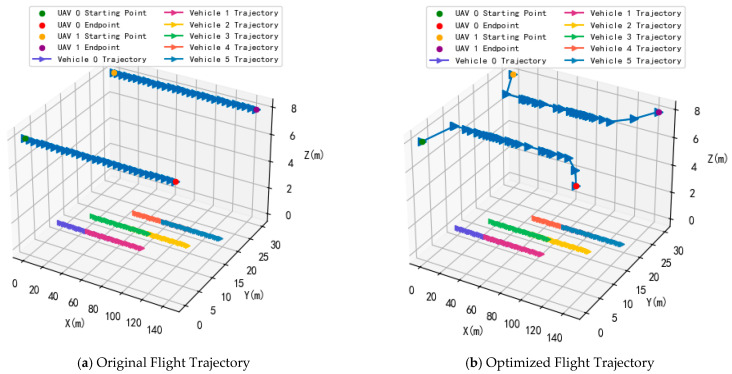
The flight trajectory of UAVs.

Figure 4 shows a comparison of the initial UAV trajectory state and the optimized UAV trajectory state after applying the proposed algorithms. As shown in the figure, the experiment is conducted on a three-lane simulation highway that is 150 m long and 30 m wide, with the y-coordinates of the three lanes set at 10, 15, and 20, respectively. 6 vehicles are set to travel at a constant speed on the three lanes of the simulation area. In Figure 4a, two UAVs fly at a constant speed along a straight line from the starting point to the ending point on both sides of the highway along the predetermined route. In Figure 4b, the flight trajectories of two UAVs are optimized based on original trajectory using proposed algorithm. As shown in the figure, after optimization, the flight trajectories of two UAVs are closer to the vehicles traveling on the three lanes in the experimental scene. This is because distance plays a crucial role in influencing the communication rate. The shorter the distance between the UAV and the vehicle, the higher the downlink communication rate from the UAV to the vehicle. Additionally, the UAV’s flight trajectory gradually approaches the original trajectory from around 117 m on the *x*-coordinate. The main reason is that the UAV needs to reach the flight endpoint while considering energy consumption constraints and also provide the maximum downlink communication rate for the vehicles.

**Figure 5:**
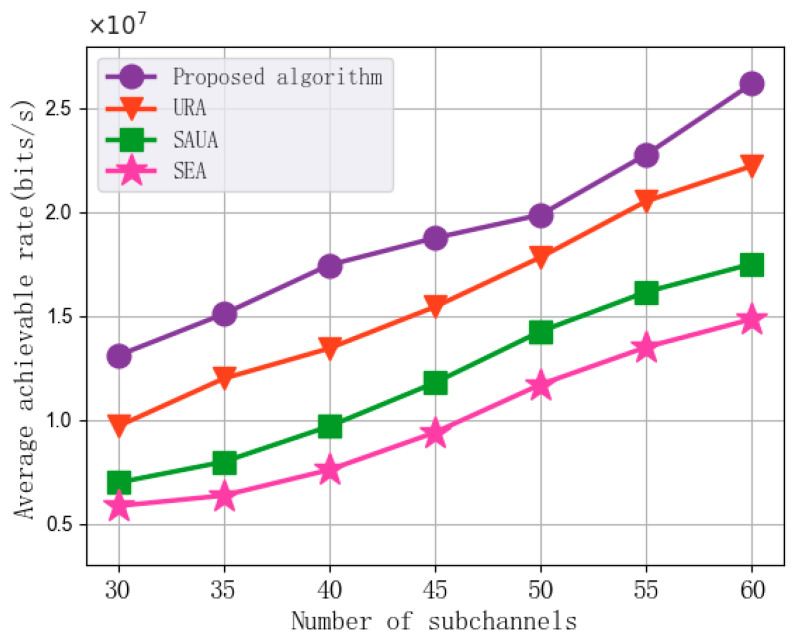
Average achievable rate vs. Number of subchannels.

Figure 5 shows the experimental results of the impact of changes in the number of subchannels on the average achievable rate performance of the algorithms. In each set of experiments, the number of subchannels is set to 30, 35, 40, 45, 50, 55, and 60, with the UAV transmission power at 2 W, the subchannel bandwidth at 1 MHz, the maximum UAV flight speed at 10 m/s, and the minimum MI requirement at 200 bits. As shown in the figure, the average achievable rate of the algorithm increases with the increase in the number of subchannels. This is because that an increase in the number of subchannels allows more subchannels to be allocated for communication functions while meeting the radar sensing requirements. This increases the communication bandwidth between vehicles and edge nodes, thereby enhancing the downlink data rate. Compared with URA, SAUA, and SEA, the proposed algorithm achieves an average improvement of 19.75%, 57.37%, and 92.11% in average achievable rate, respectively. This indicates that as the number of subchannels in the system increases, the proposed algorithm effectively utilizes the additional subchannels by reasonably allocating subchannel resources and combining the optimization of vehicle association and UAV trajectory planning, thereby achieving better data transmission rate performance than the comparison algorithms.

**Figure 6:**
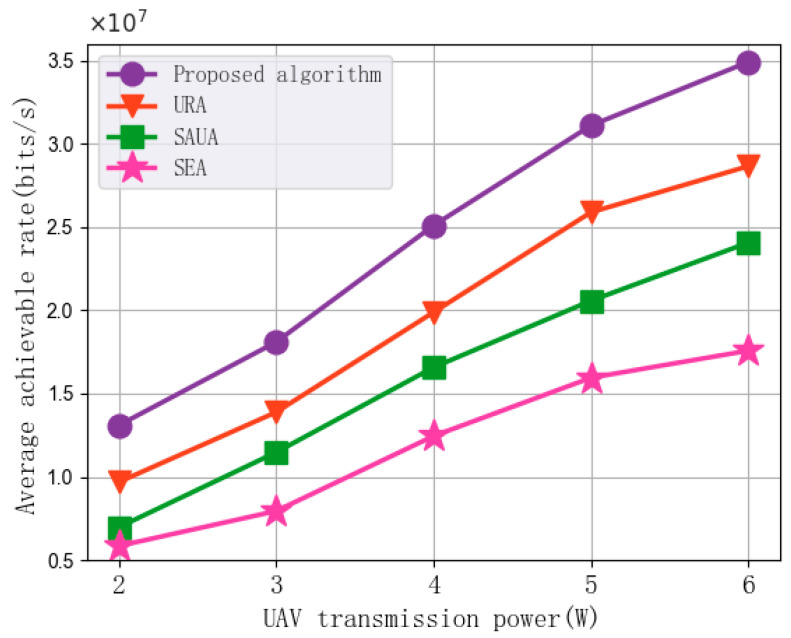
Average achievable rate vs. UAV transmission power.

Figure 6 shows the experimental results of the impact of changes in UAV transmission power on the average achievable rate of the algorithms. In each set of experiments, the UAV transmission power is set to 2 W, 3 W, 4 W, 5 W, and 6 W, with the number of subchannels set to 30, the subchannel bandwidth at 1 MHz, the maximum UAV flight speed at 10 m/s, and the minimum MI requirement at 200 bits. As shown in the figure, average achievable rate performance increases with the increase in UAV transmission power. The reason is that within a certain range, the transmission power is directly proportional to the communication rate and MI, which is consistent with the patterns described by mathematical models (11), (13), (18), and (19). Simultaneously, as the transmission power increases, MI also increases, which allows more subchannel resources to be allocated to enhance the downlink communication rate performance. This results in an increase in the average achievable rate performance. Compared with URA, SAUA, and SEA, the proposed algorithm achieves an average improvement of 24.08%, 53.73%, and 104.05% in average achievable rate, respectively. This suggests that the proposed algorithm is capable of sustaining a higher average achievable rate even as transmission power varies.

**Figure 7:**
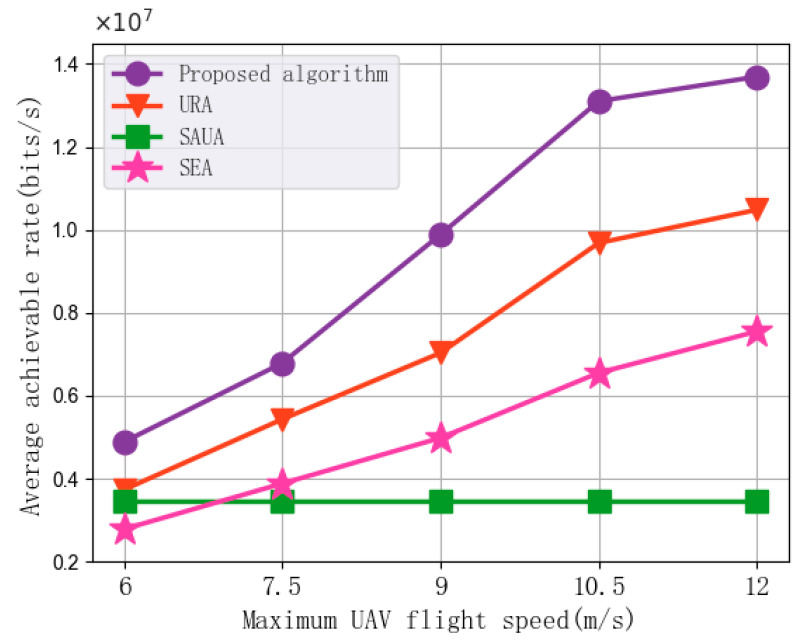
Average achievable rate vs. Maximum UAV flight speed.

Figure 7 shows the impact of changes in the maximum UAV flight speed on the average achievable rate of the algorithms. In each set of experiments, the maximum UAV flight speed is set to 6 m/s, 7.5 m/s, 9 m/s, 10.5 m/s, and 12 m/s, with the number of subchannels at 30, the UAV transmission power at 2 W, the subchannel bandwidth at 1 MHz, and the minimum MI requirement at 200 bits. As shown in the figure, the average achievable rate increases with the increase in the maximum UAV flight speed. The main reason is that an increase in UAV flight speed allows the optimization algorithm to position the UAV in more optimal locations within each time slot, thereby improving the efficiency of UAVs providing downlink communication for vehicles. It can be noted that the average achievable rate performance of SAUA does not change with the UAV flight speed. The reason is that during the experiment, SAUA does not optimize the UAV trajectory, and UAVs flies along a predetermined route. Compared with URA, SAUA, and SEA, the proposed algorithm achieves an average improvement of 32.42%, 178.57%, and 87.01% in average achievable rate, respectively. The main reason is that the proposed algorithm can efficiently utilize the increased UAV flight speed by jointly optimizing resource allocation and UAV trajectory planning. This allows the UAV to move to positions in each time slot that maximize the downlink communication rate, thereby enhancing the average achievable rate performance. 

**Figure 8:**
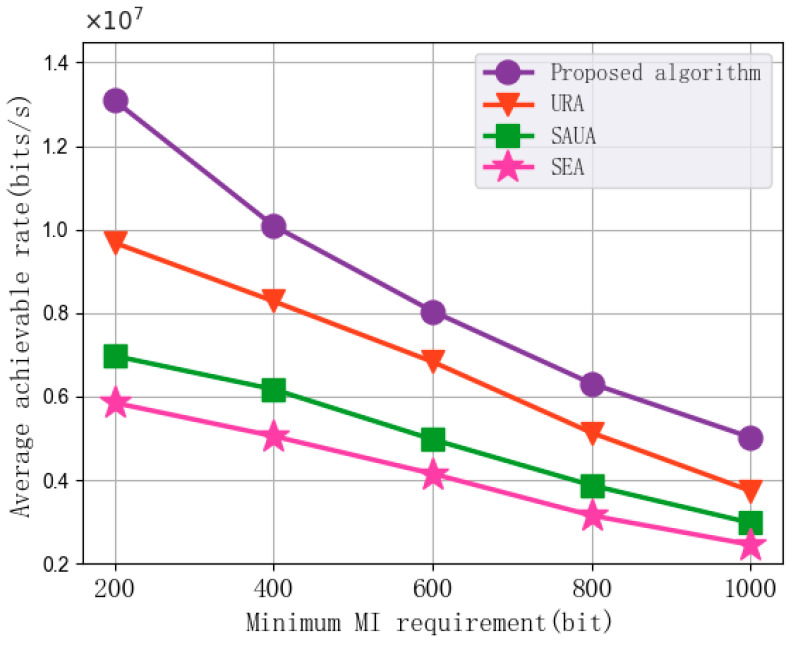
Average achievable rate vs. Minimum MI requirement.

Figure 8 shows the impact of changes in the vehicle’s minimum MI requirement on the average achievable rate performance of the algorithms. In each set of experiments, the minimum MI requirement is set to 200 bits, 400 bits, 600 bits, 800 bits, and 1000 bits, with the number of subchannels at 30, the UAV transmission power at 2 W, the subchannel bandwidth at 1 MHz, and the maximum UAV flight speed set to 10 m/s. As can be seen from the figure, the average achievable rate performance of the algorithm decreases with the increase in the minimum MI requirement. The main reason is that an increase in the minimum MI requirement leads to more subchannels being allocated for radar sensing functions, reducing the number of subchannels available for communication functions, thereby lowering the downlink communication rate. Additionally, compared with URA, SAUA, and SEA, the proposed algorithm achieves an average improvement of 26.03%, 71.10%, and 105.63% in average achievable rate, respectively. This indicates that the proposed algorithm can effectively utilize the limited subchannel resources to enhance the average achievable rate performance when communication resources are constrained, by jointly optimizing UAV trajectory planning and vehicle association and reasonably allocating subchannels. 

The MI-based sensing constraint plays a direct role in shaping the communication resource allocation decisions in the TDRA framework. When the MI threshold increases, the UAV is required to maintain trajectory segments that are closer to the sensing-favorable region in order to guarantee sufficient echo quality. This restriction limits the ability of the UAV to move freely toward communication-optimal positions. Likewise, stricter sensing constraints reduce the flexibility of the subchannel and power allocation process, as additional communication resources must be dedicated to maintaining the required sensing performance. This behavior is consistent with the fundamental sensing–communication tradeoff widely reported in ISAC literature [52]. As shown in Figure 8, the achievable communication rate decreases monotonically as the minimum MI requirement increases. When the sensing constraint becomes more stringent, the optimization prioritizes sensing feasibility, leading to reduced communication throughput. These observations quantitatively demonstrate how the MI constraint affects the trajectory planning and resource allocation decisions, revealing the inherent tradeoff structure in UAV-assisted vehicular ISAC systems.

**Figure 9:**
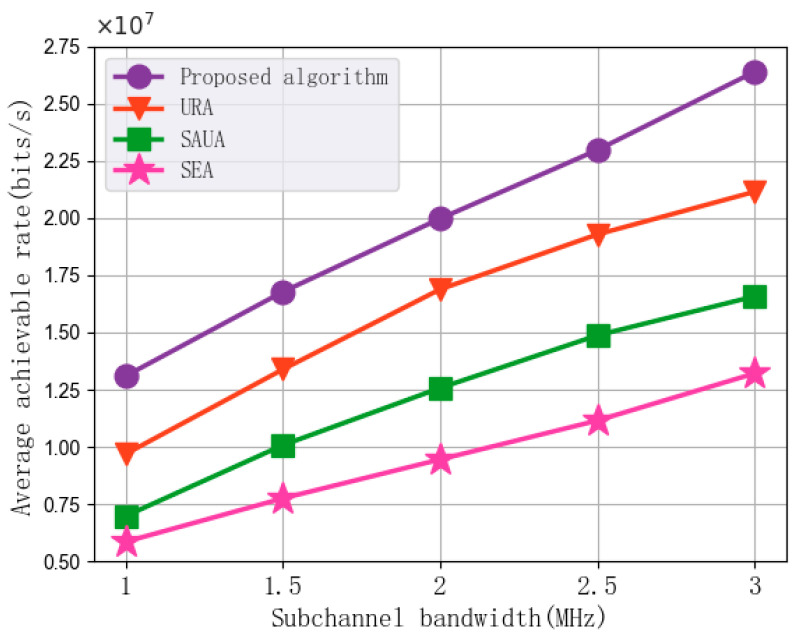
Average achievable rate vs. Subchannel bandwidth.

Figure 9 shows the experimental results of the impact of changes in subchannel bandwidth on the average achievable rate performance of the algorithm. In each set of experiments, the subchannel bandwidth is set to 1 MHz, 1.5 MHz, 2 MHz, 2.5 MHz, and 3 MHz, with the number of subchannels at 30, the UAV transmission power at 2 W, the maximum UAV flight speed set to 10 m/s, and the minimum MI requirement at 200 bits. As can be seen from the figure, the average achievable rate performance of the algorithms increases with the increase in subchannel bandwidth. The main reason is that an increase in subchannel bandwidth allows vehicles allocated the same number of subchannels to have a higher downlink transmission rate, thereby enhancing the average achievable rate performance. Compared with URA, SAUA, and SEA, the proposed algorithm achieves an average improvement of 22.71%, 62.29%, and 109.92% in average achievable rate, respectively. This demonstrates the effectiveness of the proposed algorithm. 

In addition, the simulation results in Figure 5, Figure 6, Figure 7, Figure 8 and Figure 9 demonstrate the robustness of the proposed algorithm. Although key system parameters such as UAV speed, transmission power, subchannel bandwidth, and minimum MI requirement vary over a wide range, the proposed algorithm consistently maintains stable convergence behavior and superior performance compared with all baseline methods. Moreover, the algorithm consistently achieves significantly higher performance than the benchmarks in Figure 5, Figure 6, Figure 7, Figure 8 and Figure 9, indicating that the approximation error introduced by the first-order Taylor expansion does not degrade the final solution quality. These results validate the reliability of the SCA-based approximation and confirm that the BCD–SCA framework remains robust under diverse parameter settings and external uncertainties.

## 7. Conclusions

To address the issue of decreased efficiency in providing communication and radar sensing services in the ISAC-enabled UAV-assisted vehicular network architecture due to limited spectrum resources, mutual constraints between communication and sensing resources, and unreasonable UAV trajectory planning, this paper proposes a TDRA strategy. The strategy constructs a resource allocation model for communication and radar sensing. By jointly optimizing UAV trajectory planning, vehicle association, and subchannel allocation, an optimization problem is formulated to maximize the average achievable rate, taking into account constraints such as UAV energy consumption and radar sensing performance. Then, a block coordinate descent-based iterative algorithm is designed to solve the optimization problem iteratively, yielding an approximate optimal solution. The experimental results demonstrate that the proposed algorithm can effectively enhance average achievable rate performance. 

While the proposed TDRA strategy is evaluated under idealized channel and mobility assumptions, several practical factors may affect its real-world deployment. First, channel estimation errors may degrade the accuracy of both communication-rate evaluation and MI-based sensing constraints. Incorporating robust resource allocation or uncertainty-aware sensing models could mitigate this limitation. Second, mobility prediction inaccuracies for vehicles or UAVs may lead to suboptimal trajectory planning, especially in highly dynamic environments. Future extensions could integrate learning-based mobility prediction or real-time trajectory correction. Third, the TDRA algorithm introduces computational overhead associated with solving the BCD–SCA subproblems. Although the algorithm converges within a few iterations in our simulations, real-time implementations may require further acceleration via parallelization, model simplification, or lightweight approximation methods. Addressing these aspects represents an important direction for practical ISAC-enabled vehicular deployments.

## Figures and Tables

**Table 1:** System parameters.

**Parameters**	**Definition**
Number of vehicles *V*	6
Number of UAVs *U*	2
Number of GBSs *R*	1
Number of sub-channels *C*	30–60
Fixed altitude of UAVs $h_{u}$	8 m
Maximum flight speed of UAVs $md_{u}^{\max}$	6–12 m/s
Minimum safe distance between UAVs $d_{\min}$	8 m
Energy required for UAV to fly a unit distance $sp_{u}$	2 J/m
Duration between time slots τ	1 s
Number of time slots contained in the time period *T*	30
Simulation scene size	150 m × 30 m
Channel power gain at a reference distance of 1 m $h_{v,u}^{c}$, $h_{v,r}^{c}$	−60 dB
Gaussian white noise power $N_{0}$	−145 dBm
Subchannel bandwidth $B_{o}$	1–3 MHz
On-board energy of UAVs $EC_{u}^{\max}$	500 J
Transmission power of UAV $P_{u}^{t}$	2–6 W
Transmission power of GBS $P_{r}^{t}$	3 W
Basic communication rate requirements for vehicle $TR_{v}^{req}$	2 × 10^4^ bit/s
Minimum MI requirement threshold of the vehicle $HMI_{v}^{t}$	200–1000 bit
Noise power on the *c*-th subcarrier $\sigma_{u,rad}^{c}$, $\sigma_{r,rad}^{c}$	1.66 × 10^−14^ W
Number of consecutive OFDM symbols *S*	10
Duration of the OFDM symbol $T_{s}$	5 µs

## Data Availability

Dataset available on request from the authors.
